# A versatile hybrid agent-based, particle and partial differential equations method to analyze vascular adaptation

**DOI:** 10.1007/s10237-018-1065-0

**Published:** 2018-08-09

**Authors:** Marc Garbey, Stefano Casarin, Scott A. Berceli

**Affiliations:** 1 Houston Methodist Research Institute, Houston, TX USA; 2 Department of Surgery, Houston Methodist Hospital, Houston, TX USA; 3 LaSIE, UMR CNRS 7356, University of la Rochelle, La Rochelle, France; 4 Department of Surgery, University of Florida, Gainesville, FL USA; 5 Malcom Randall VAMC, Gainesville, FL USA

**Keywords:** Vascular adaptation, Particle model, IBM method, PDE model

## Abstract

Peripheral arterial occlusive disease is a chronic pathology affecting at least 8–12 million people in the USA, typically treated with a vein graft bypass or through the deployment of a stent in order to restore the physiological circulation. Failure of peripheral endovascular interventions occurs at the intersection of vascular biology, biomechanics, and clinical decision making. It is our hypothesis that the majority of endovascular treatment approaches share the same driving mechanisms and that a deep understanding of the adaptation process is pivotal in order to improve the current outcome of the procedure. The postsurgical adaptation of vein graft bypasses offers the perfect example of how the balance between intimal hyperplasia and wall remodeling determines the failure or the success of the intervention. Accordingly, this work presents a versatile computational model able to capture the feedback loop that describes the interaction between events at cellular/tissue level and mechano-environmental conditions. The work here presented is a generalization and an improvement of a previous work by our group of investigators, where an agent-based model uses a cellular automata principle on a fixed hexagonal grid to reproduce the leading events of the graft’s restenosis. The new hybrid model here presented allows a more realistic simulation both of the biological laws that drive the cellular behavior and of the active role of the membranes that separate the various layers of the vein. The novel feature is to use an immersed boundary implementation of a highly viscous flow to represent SMC motility and matrix reorganization in response to graft adaptation. Our implementation is modular, and this makes us able to choose the right compromise between closeness to the physiological reality and complexity of the model. The focus of this paper is to offer a new modular implementation that combines the best features of an agent-based model, continuum mechanics, and particle-tracking methods to cope with the multiscale nature of the adaptation phenomena. This hybrid method allows us to quickly test various hypotheses with a particular attention to cellular motility, a process that we demonstrated should be driven by mechanical homeostasis in order to maintain the right balance between cells and extracellular matrix in order to reproduce a distribution similar to histological experimental data from vein grafts.

**Electronic supplementary material:**

The online version of this article (10.1007/s10237-018-1065-0) contains supplementary material, which is available to authorized users.

## Introduction and motivation

The insurgence of an arterial localized occlusion, known as peripheral arterial occlusive disease (PAOD), is one of the potential causes of tissue necroses and organ failure, and it represents one of the main causes of mortality and morbidity in the western society (Go et al. 2014; Roger et al. 2012).

In order to restore the physiological circulation, the most performed technique consists into bypassing the occlusion with an autologous vein graft. Benefits and limitations of this procedure are driven by fundamental mechano-biology processes that take place immediately after the surgical intervention and that fall under the common field of vascular adaptation. The plasticity of the biological system is indeed responsible for the adaptation of the vein to its new environment, and it is mostly driven by two distinct processes: intimal hyperplasia and wall remodeling, this latter either inward or outward.

If on the one end, intimal hyperplasia is characterized by migration of smooth muscle cells (SMCs) into the intima with subsequent cells proliferation and deposition of extracellular matrix (ECM), typically favoring the lumen narrowing, on the other end wall remodeling is characterized by the preservation, or the loss, of lumen area through reorganization of the cellular and extracellular components within the media (de Korte 2000; Lytle et al. 1985; Saswata et al. 2013; Owens 2010).

Starting from the very early postsurgical period, both the processes are initiated and it is the balance between them that dictates the degree of luminal narrowing and ultimately the success or failure of the revascularization (Owens 2010; Chiu and Chien 2011).

Despite years of clinical research resulting in significant improvements in the surgical techniques, the rate of failure of this procedure is of 12% after just a month from the original intervention, a percentage that ramps up to 40% after 10 years (Conte et al. 2006; Alexander et al. 2005). Early vein graft remodeling is believed to be induced by hemodynamic forces, where wall shear stress stands among the primary regulators for these events (Varty et al. 1993; Mills et al. 1995). However, the biological mechanisms under vein graft failure are not completely understood and in addition several clinical observations have shown how such failure events are difficult to predict. It is so our hypothesis that their causes are multiscale and multifactorial, and accordingly it would be reductive and also wrong to just resume them into some basic explanations related to the local variation of shear stress or wall pressure (Jiang et al. 2004; Dobrin et al. 1989; Fillinger et al. 1994; Zwolak et al. 1987; Galt et al. 1993).

This multiscale character naturally extends to the vascular adaptation itself: The localized variation of hemodynamics conditions is the trigger that initiates a cascade of events that drive tissue remodeling. Further, a variation in environmental conditions, such as in shear stress, modifies the working point of the gene regulatory network, which dictates the cell and matrix-based remodeling response, defining in this way the local geometrical modifications. Finally, the morphological changes induce perturbations in local shear stress, resulting in a new set point of the gene network, and consequently in a new biological response of the graft.

**Fig. 2:**
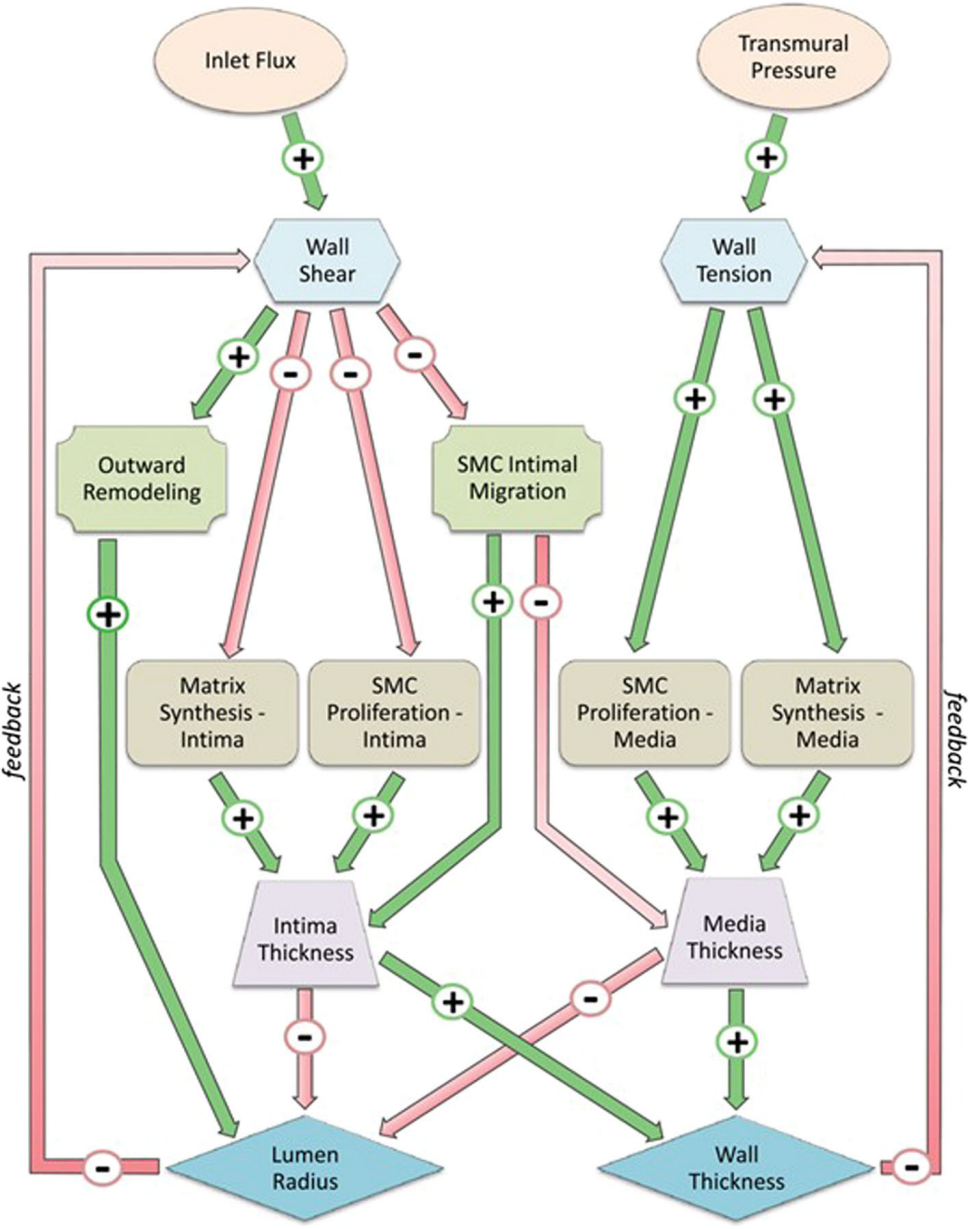
Conceptual scheme Parallel cascade of events following hemodynamics conditions variations. Connections between nodes are expressed by an oriented link in the graph, where $A \rightarrow B$ represents a direct influence of node A on node B. If A induces an increase of B, the corresponding linkage is indexed with a plus sign. Alternatively, if A induces a reduction of B, the corresponding linkage is negative (Garbey and Berceli 2013)

The conceptual scheme of Fig. 1 gives a better inside on the multiple feedback loops that link wall shear stress and wall tension variations with morphology changes at tissue level through specific cellular and ECM-related dynamic (Garbey and Berceli 2013). The presented scheme has been translated into a dynamical system (DS) developed in a previous work by our group of investigators (Garbey and Berceli 2013) showing how different feedback cycles can either promote the same outcome or even compete by promoting opposite adaptive mechanisms. However, such study is phenomenological by nature and it lacks the level of understanding needed to analyze spatiotemporal behavior of SMCs appreciable by histological data, an example of which is given in Fig. 2a that refers to a vein graft at the time of implantation and in Fig. 2b that instead refers to two different outcomes after a 6-month postsurgical follow-up.

Following the general concepts discussed in Evans et al. (2008) and Berceli et al. (2009), the goal of the present work is to address the modeling and simulation of the vascular adaptation processes from a multiscale perspective that has already proved itself to be effective if used to study the cardiovascular system (Zhang et al. 2016). By using this approach, a virtual experimental framework is provided in order to be used to test new clinical hypotheses and to better rank the influence of the many factors that may or may not promote restenosis.

This paper is based on the extensive work carried out by our group on vascular adaptation that extends from clinical data analysis to computational modeling (Hwang et al. 2013; Garbey et al. 2015; Garbey and Berceli 2013; Garbey et al. 2017; Casarin et al. 2017). Thanks to a more effective choice of the support structure, the work presented can take into account pivotal biological events such as cellular motility and cell–cell, cell–membrane interactions that were very difficult to replicate with a standard discrete agent-based model (ABM) on a fixed grid (Garbey et al. 2015, 2017), getting in this way closer to the physiological reality and representing a more reliable platform to study the restenosis phenomenon.

**Fig. 3:**
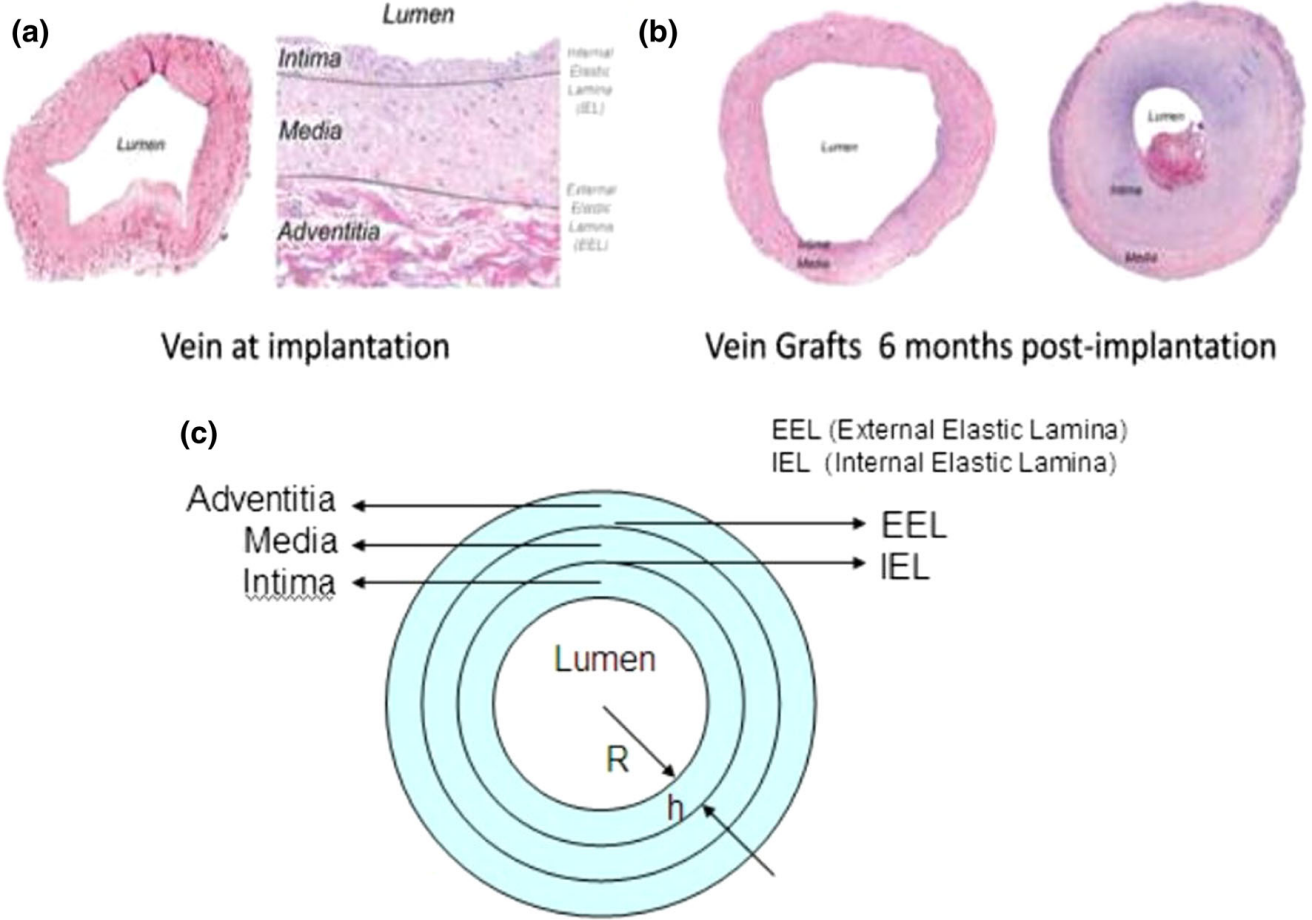
Vein graft histology and anatomy histological measurements conducted both at the time of graft implantation, also pointing out the separation between intimal and medial layers (**a**), and after a 6-month post-implantation follow-up (**b**) with a comparison between two types of outcome, clearly an effective remodeling (left) against a re-occlusion of the graft (right). To be underlined how markedly different phenotypes ranging from preservation of the lumen to severe intimal thickening and lumen narrowing are commonly encountered (Garbey and Berceli 2013). The schematic cross section of a vein graft (**c**) finally shows how between the intima and the media is the internal elastic lamina (IEL), while the external elastic lamina (EEL) is between media and adventitia

As a proof of concept, the hyperplasia of the tunica intima is replicated with our new model on a two-dimensional cross section of the vein graft. The choice of a 2D geometry is in accordance with the nature of our experimental setup on rabbit model, described in Fig. 3, which provides that wall thickening is almost independent from the third dimension.

The goal of this paper is to show how a detailed description of the different potentials driving cellular motility during adaptation is the key to obtain a model close enough to the physiological reality. We chose as reference histological data from our already-cited experimental model of rabbit, and we set as goal to be able to reach the same uniform distribution of cells against ECM typically appreciable from histology. In addition, the choice to qualitatively validate the model against histological data, available in the format of 2D slices, further corroborates our choice of a 2D approximation.

Nonetheless, the extension of the model to a 3D space does not pose in principle any particular issue. It would just require a fairly large investment in terms of software development and parallel computing. Once the model will be escalated to the understanding of the effect of local curvatures and/or bifurcation, a 3D computational model will be required, but this is not the case for the current work.

Finally, the new model has been cross-validated against the DS and the ABM previously cited. This is an important step of the development of every model; indeed, as largely discussed in Garbey et al. (2017), to be able to cross-validate two computational frameworks allows to choose the best model to be used according to the purpose of the analysis without giving up on the models’ accuracy.

## Methods

In order to replicate the anatomy of a vein graft’s cross section, shown in Fig. 2c, the support structure of the computational model is divided in four sub-domains, which are lumen, tunica intima, tunica media, and external surrounding tissue (external support). The intima is separated from the media by a so-called internal elastic lamina (IEL) while the adventitia is neglected being typically removed during surgery.

**Fig. 4:**
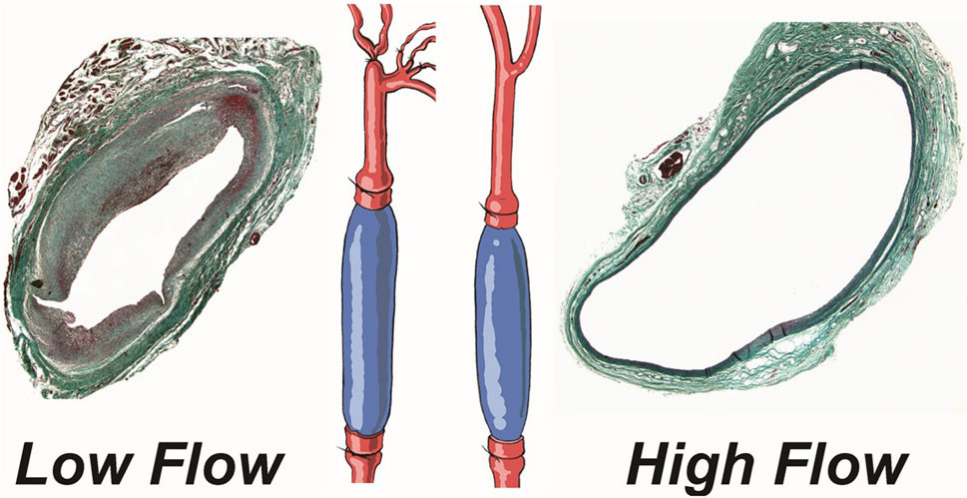
Experimental model bilateral vein graft with distal branch ligation model. The most inferior branch of the external carotid served as the only outflow for the low flow graft on the ligated side, resulting in two distinct flow regimes (Klein et al. 2017)

The numerical model can be decomposed into three sub-modules:

- Mechanical model (**MM**): It locally computes the value of the mechanical quantities of interest such as luminal blood flow velocity, strain energy across the wall, shear stress at the wall, transmural pressure as well as diffusion of growth factors (GFs) inside the wall.
- Tissue plasticity (**TP**): It defines the SMC mitosis/apoptosis and ECM deposition/degeneration performed by SMCs as stochastic laws driven by constant coefficients.
- Tissue remodeling (**TR**): It computes the cellular migration that determines the matrix reorganization. This step is the real core of the vascular adaptation, and it represents the key to finely replicate the inward/outward tissue remodeling.

The general rationale of our method sees that the MM is best described by the well-known partial differential equations (PDEs) of continuous mechanic described in White (1991), the TP by an ABM that describes individual cells behavior (Garbey et al. 2015, 2017), and the TR by particles moving in a highly viscous incompressible media. Further on the latter, the new way TR is based on the concept that cells crawl through and within the ECM regulated by a variety of mecano-biology stimuli, and accordingly cells’ motion should be computed on the basis of a continuum space (Quaranta 2000) rather than on a discrete grid.

The most challenging part is to be able to finely model the forces that drive cellular motility both in a realistic way that fits biological observations, but also with a relatively simple mathematical formula that can be easily calibrated on experimental data. As highlighted in Introduction, the cornerstone of our model is its multiscale nature and so its building must encompass multiple scales both in time and in space, as described in Table 1.

**Table 1:** Multiscale nature of the hybrid model

Space scale versus timescale	Second	Hour	Day
$10^{-4}\, \hbox {m}$		TR	TP
$10^{-3}\, \hbox {m}$	CM	TR	
$10^{-2}\, \hbox {m}$	CM		

The numerical discretization and the algorithm implemented for each module and the sub-modules coupling mechanisms share the same level of importance, and they will be described in the following subsections.

### Mechanical model (MM)

The blood flow in the lumen is described as a steady incompressible flow that remains constant independently from the inward or the outward nature of the graft remodeling. This choice is in accordance with the property of the vascular system of having a global controlled mechanism to maintain a constant blood flow delivery (Guyton and Hall 2000).

This mechanism is responsible for the fact that often stenoses are diagnosed only when the occlusion of the vessel is so severe that makes this control mechanism to fail. Accordingly, the numerical simulation was here implemented in order to arrest once a stenosis has reached a 50% occlusion.

The timescale for tissue plasticity, typically from days to weeks, is sufficiently larger than the cardiac pulsation’s frequency (order of seconds) to assume that the cells adapt in response to a time-averaged condition of the mechanical environment. We so implicitly supposed that the error induced by neglecting the nonlinear convective term in the Navier–Stokes equations and the use of a steady flow solution is smaller than the level of uncertainty that characterizes the biology of the system. As a result, the standard set of equations of a fully developed flow through a pipe was used to simulate the blood flow across the vein, also assuming a nonslip condition at the wall (White 1991; Maas et al. 2012).

The MM computes the velocity field inside the lumen and the shear stress at the wall, labeled as $\tau _{\mathrm{wall}}$, and both the variables are updated if the variation of lumen geometry from one time step to the subsequent is greater than a certain tolerance. The variation of lumen geometry is defined as follows:

1 $$\begin{aligned} \mathrm{distance}\left( \partial \varOmega _{\mathrm{lumen}}^{\mathrm{new}},\partial \varOmega _{\mathrm{lumen}}^{\mathrm{old}}\right) , \end{aligned}$$

where $\mathrm{distance}$ is the Euclidean distance between two consecutive time points of the lumen and $\mathrm{tol}$ is of the order of an SMC diameter.

As this condition implies more than just one cycle of cell division to be satisfied, it is clear how the mechanical properties of the vein need to be updated only every few hours because of the timescale of cell division and this is in accordance with the steady flow approximation used for this model.

The deformation of the wall was described either with a thick cylinder approximation that can be easily computed analytically with a MATLAB code (Zhao et al. 2003), or by a neo-Hookean hyperelastic model, which is instead computed by using a finite elements technique with FEBio software (Maas et al. 2012). As guideline, the choice is driven by the degree of closeness between wall and cylinder shape, for which the closer they are, the more acceptable is to use a simple thick cylinder approximation.

The description of the tissue mechanical properties is the one already adopted in previous works by our group of investigators (Garbey and Berceli 2013; Garbey et al. 2015), and accordingly, while the displacement of the wall is relatively negligible, the spatial distribution of strain energy in the wall, denoted as $\sigma$, influences SMCs’ metabolism within the media. Additional details are also provided in “Appendix 1” section.

Finally, the diffusion of a generic SMC division growth factor (GF), triggered by the shear stress across the wall, and so denoted as $G(\tau )$, is computed with a standard diffusion problem as follows:

2 $$\begin{aligned} \frac{\partial G}{\partial t} = c \; \Delta G \; \hbox {in} \; \varOmega , \quad G_{|\partial \varOmega _{\mathrm{lumen}}} = F(\tau _{\mathrm{wall}}), \quad {\frac{\partial G}{\partial n}} \; {|\partial \varOmega }=0, \end{aligned}$$

where *c* is the diffusion coefficient. The use of GF is driven by the need of transferring the biological effect of the shear stress inside the wall.

In order to maintain the simplicity of the implementation, both the flow solver and the diffusion operator are implemented with finite differences technique on a regular cartesian grid with a space step slightly smaller than a SMC diameter. As a first approximation, we used standard isotropic diffusion for this 2D approximation of the wall. Finally, the Dirichlet boundary conditions at lumen wall and the Neumann boundary conditions at the external wall are imposed by penalty method (Angot et al. 1999; Angot 2005).

### Tissue plasticity (TP)

The activity of SMCs and ECM is described with an ABM-based implementation (Wolfram 1983; Deutsch and Dormann 2005; Hwang et al. 2013) that is mostly based on a cellular automata principle governed by stochastic laws. Accordingly, each cellular event is associated with a density of probability, represented by a mathematical function. “Appendix 2” section gives a better insight on the ABM, and the set of functions implemented in the current model is summarized in Table 2 that reports the densities of probability for SMC mitosis/apoptosis and ECM synthesis/degeneration.

**Table 2:** Axiomatic description of the set of rules of the ABM

Rule	Variable	Function
$p_{\mathrm{division}}=p_{\mathrm{apoptosis}}=\alpha _1,$	SMC	SMC equilibrium in basic solution
$p_{\mathrm{degradation}}=p_{\mathrm{production}}=\alpha _2,$	ECM	ECM balance in basic solution
$A(t)= \exp {-\frac{t-T}{\delta T}}; \; T=\alpha _3, \; \delta T=\alpha _4$	All	Factor all probability laws by macrophage activity
*T* and $\delta T$	Macrophage	time of maximum macrophage activity and relaxation time
$p_{\mathrm{division}}^{I}=\alpha _1 A(t) \left( 1+\alpha _5 \frac{G(\Delta \tau )}{\bar{\tau }}\right)$	SMC	Probability of SMC division in intima
$p_{\mathrm{apoptosis}}^{I}=\alpha _1 A(t)$	SMC	Probability of SMC apoptosis in intima
$p_{\mathrm{production}}^{I}=\alpha _2 A(t) \left( 1+\alpha _6 \frac{\Delta \sigma }{\bar{\sigma }}\right)$	ECM	Probability of ECM production in media
$p_{\mathrm{degradation}}^{I}=\alpha _2 A(t)$	ECM	Probability of ECM degradation in media
$p_{\mathrm{migration}}=\alpha _7 A(t) \left( 1+\alpha _8 \frac{G(\Delta \tau )}{\bar{\tau }}\right)$	SMC	Probability of SMC migration from intima to media

The stochastic model describes how specific cellular events depend on the local concentration of the associated GF that is itself triggered by low shear stress at intimal level or high strain energy at medial level (Szilagyi et al. 1973; Roddy et al. 2003), making the probability of a cellular event shear dependent and so, transitively, space dependent.

Intimal hyperplasia is the quasi-universal response to a vascular injury for which a reduction of shear stress stimulates specific GFs to switch their status from quiescent to active. In order to simulate the switching from a normal condition to a perturbed one, the key is to define a so-called basic solution, where the system is stable and it is regulated by standard mechano-environmental conditions that ensure a fair balance between SMC rate of mitosis and apoptosis and again a fair balance between the rate of ECM synthesis and degradation.

To simulate both the basic solution and the perturbed state, a mitotic cycle of 12 h has been chosen along with a 2 h cycle for the ECM synthesis/degradation by a SMC. The choice of both the mitotic cycle and the ECM synthesis time is in accordance with previous works by our group of investigators (Garbey et al. 2015); however, a better study of the mitotic cycle length might further improve the accuracy of the model. It is to be expected that said time is closer to 24 h than 12 h, even though, seen the current implementation of the model, a 12 h difference does not bring to a significant difference. The system is investigated with a time step of 1 h, in which each site of the ABM is interrogated to define if it is active or not.

The basic solution truly represents a “healthy” vein at the time of implant, and all the probability laws will be expressed as function of the deviation of shear stress or strain energy from an ideal shear/tension condition reached with the basic solution itself.

To simulate the hyperplasia of the intima, it has to be remarked how, when the vein graft is exposed to low shear stress, the probability of SMC division is promoted in the intimal layer and in particular in the spatial area next to the lumen. This displacement in SMC production decreases while moving away from the lumen wall also depending on the rate of decay of the GF concentration through the wall thickness that depends on the diffusion coefficient *c*, as described with (2).

Finally, the rationale and the experimental background behind the formulation of the probabilities of Table 2 have been already largely addressed by our group in Garbey et al. (2015) and Garbey et al. (2017) as well as the testing and the cross-validation of the TP model (Garbey et al. 2017).

### Tissue remodeling (TR)

In our previous works (Garbey et al. 2015, 2017), a single SMC or an ECM element was represented by a hexagonal site on a fixed grid and the reorganization of the matrix in response to the graft’s adaptation was addressed by discretely shifting the structure site by site following a minimum energy path motion.

The flaw of this implementation consists in the fact that a fixed grid constrains the reorganization of the matrix as the tissue remodels itself.

It is for this reason that the greatest novelty of the current approach is represented by the choice of a continuous mechanic description for the structure that the model lays on that is believed to have a better potential to represent the biology accurately. Specifically, SMCs are not anymore described as a hexagonal element of a fixed grid, but as particles crawling in a highly viscous flow, representing the suspension matrix, and modeled as a simple disc of radius $R_{\mathrm{SMC}}$.

According to biological evidences, SMCs can generate or also degrade elements of ECM and this represents, respectively, a source and a sink term in the balance of mass that will be used to minimize the energy of the structure. Finally, as described in Fig. 2c, each layer of the graft is bounded by an elastic membrane.

The ensemble of these considerations naturally suggests the use of the immersed boundary technique originally developed by Peskin (2002) in order to simulate the remodeling of the vascular structure.

The tissue remodeling is articulated in three phases that will be addressed separately in the next subsections:

1. The immersed boundary method (IBM) algorithm and its volume correction to take into account both the cell activities and the membrane motion adjustment
2. The algorithm to describe SMCs motion under cell–cell repulsion and other additional external forces
3. The inward versus outward remodeling at each time step that will be chosen to minimize the mechanical energy of the wall.

#### IBM algorithm

The tissue remodeling is achieved by running the IBM algorithm for a period of time $\delta t$ that corresponds to the tissue relaxation time itself.

A time-split numerical implementation was considered for which, at every cycle, the TP model is run with a time step of 1 h, while the time step for the IBM algorithm, $\delta t$, is an unknown parameter of the model, and it is legit to expect that the larger $\delta t$, the more cylindrical the vein graft will end to be.

On the other end, the spatial discretization of the IBM is a Cartesian grid of space step *h* in both directions, where *h* is chosen of the order of the SMC radius.

The formulation of the IBM algorithm is based on the definition of the domains that it lays on and of the variables that is regulated by. The algorithm is described in detail in “Appendix 3” section, and in addition to it, the IBM algorithm (Peskin 2002) offers dozen of different possibilities regarding the choice of the temporal scheme, the space discretization, the discrete approximation of the Dirac function, et cetera.

While planning the implementation, a compromise between the stability of the scheme, which typically suffers from the sharp numerical interface within the pressure field, and the accuracy needed by this numerical feature must be taken into account. A good example of how to manage this necessary compromise is offered by Pacull and Garbey (2009), where a comparison between possible implementations against standard benchmark problems is finely analyzed. Good examples of these problems are the oscillation/relaxation of a stretched “bubble” toward its equilibrium, or the motion of an elastic “bubble” immersed in a cavity flow.

A standard projection scheme for the Navier–Stokes equations discretized with finite differences on a staggered grid was used.

The momentum equation was discretized with central second-order finite differences for the diffusion term and with a method of characteristic for the convective term. The time stepping is semi-implicit and first order in time to compute a prediction of the velocity field $V^*$ at time $t^{n+1}$. Also, the correction step to ensure mass conservation uses the following Hodge decomposition:

3 $$\begin{aligned} V^*= & {} V^{n+1}+\frac{\Delta t}{\rho } \nabla \varPi \end{aligned}$$

4 $$\begin{aligned} \nabla \cdot V^{n+1}= & {} 0, \; V^{n+1}_{| \partial \varOmega }=0, \end{aligned}$$

where $V^{n+1}$ is the divergence-free exact projection of $V^*$ and the corresponding projection operator is:

$$\begin{aligned} \left[ I-\nabla \Delta ^{-1}\nabla \right] . \end{aligned}$$

The divergence of (3) leads to the pressure correction solution of the Poisson problem as follows:

5 $$\begin{aligned} \Delta \varPi = \frac{\rho }{\Delta t}\nabla \cdot V^* \end{aligned}$$

By modifying the right-hand-side term of the Poisson problem described with (4), the local source or sink of mass corresponding to SMC mitosis/apoptosis or ECM production/degradation was taken into account.

Finally, the variation in SMC and ECM balance clearly depends on the unit of volume of the single SMC, that is $2 \pi R_{\mathrm{SMC}}$ and on the unit of volume of the ECM element, that is $2 \pi R_{\mathrm{ECM}}$.

#### SMC motility

The second phase of tissue remodeling consists into the computation of SMCs motility. SMCs are here modeled as particles embedded in a highly viscous media, and the algorithm to compute their trajectory can be divided into two consecutive steps.

First, SMCs move passively in the matrix by following the media on the base of the local velocity field with the same numerical scheme applied to the discrete point of the immersed boundary, and second, SMCs move also actively driven by multiple potential driving forces, listed below and reported in detail in a series of published works (Carter 1967; Bray 2000; Mitchison and Cramer 1996). The power of computational models consists in their ability to perform several virtual experiments in a relatively short time, offering the possibility to test several biological hypotheses with high flexibility. In this work, we used this feature by testing the effect of several potential candidates to be the pivotal driver of cellular motility, further focusing on four of them:

- SMCs interact with each other. A description of such interactions can be based on an analogous of the Lennard-Jones potential as an initial guess. Under this hypothesis, during mitosis the two cells may separate and remain at a distance of about their diameter. This makes the two Lennard-Jones potential coefficients to be cellular size dependent. Melnikova et al. (2017) provides a detailed cell–cell interaction potential model for the artery case that will be interesting when we will have access to measurements relative to arteries in our experimental model.
- Further motion of SMCs depends on the gradient of molecules density that are the solution of a reaction–convection diffusion system. Accordingly, a generic GF has been introduced with (2) in order to describe the chemotaxis that is originated by the cited gradient.
- Cell motility has a random component that participates in their diffusion through the tissue.
- SMCs may infiltrate area free of cells to preserve the tissue integrity. This motion corresponds to a mechanical homeostasis, and it maintains a local balance between SMC and ECM distribution to keep the matrix healthy (Quaranta 2000; Humphrey et al. 2014).

To sum up, the trajectory of a SMC can be so described by tracking its position along time with the following relation:

6 $$\begin{aligned} \dot{X}=V_{\mathrm{S}} + V_{\mathrm{E}} + V_{\mathrm{G}} + V_{\mathrm{R}}, \end{aligned}$$

where *X* is the location of the single SMC.

In (6), $V_{\mathrm{S}}$ sums up the repulsive forces between SMC. The amplitude of this force decays with the distance, and in first approximation, one can assume a linear decay toward zero in $n_{\mathrm{S}}$ units expressed in cell diameter. Consequently, cell–cell interaction is only possible between elements belonging to the same subdomain, i.e., intima or media, and also interaction is not possible between cells separated by a distance larger than $2 \; n_s \; R_{\mathrm{SMC}}$, where $n_s$ has been chosen to be of the order of few units.

$$V_{\mathrm{E}}$$ sums up the attractive forces between SMC and the surrounding area occupied by the extracellular matrix. It decays linearly as for the cell interaction but in $$n_e$$ units and becomes zero above a distance of $$2 \; n_e \; R_{\mathrm{SMC}}.$$$$n_s$$ and $$n_e$$ have a great influence on the result of the simulation, and a deep analysis of them will be useful to address some open problems of the vein graft’s biology.

$V_{\mathrm{G}}$ is proportional to the gradient of *G* that is the generic GF that activates SMC proliferation.

Finally, $V_{\mathrm{R}}$ is a random vector that mimics the noisy character of cell motility. Its introduction is justified by the assumption that a cell cannot move more than a radial unit within the time step $\delta t$ of the IBM algorithm.

An important feature of the method here proposed is that it allows us to implement all these elements that are known to play a key role at biological level and to also test several combinations of them. However, compared to our previous ABM (Garbey et al. 2017), the number of unknown parameters used to describe the new cellular motility module grows proportionally with the level of closeness of the model to the physiological reality, and accordingly, a nonlinear stability analysis will be needed to find the trade-off between complexity and accuracy as already done in Garbey et al. (2015).

#### Inward–outward membrane motion adjustment

It is important to remark how, without an ad hoc adjustment, the algorithm presented would always promote outward remodeling at the expense of inward remodeling since the medium in the lumen is incompressible.

The thought behind the design of this adjustment must be driven by the need of minimizing the energy of the deforming structure during the adaptation, and accordingly a positive/negative term has been introduced in the mass balance, like already done for SMC and ECM dynamic. After all, it has been well demonstrated that biological systems respond to perturbations by minimizing the free energy level (Karl 2012). Being here the cellular dynamic the perturbation, the wall will respond by reorganizing in order to minimize its level of free energy.

Specifically, at each cycle, the mechanical potential energy of the wall is computed with the MM and the sign of the term is chosen to be concordant with the sign of the derivative that minimizes the potential energy.

The hypothesis is so that the tissue accommodates to the trans-mural pressure that is a combination of blood pressure and external pressure from the surrounding tissue toward a state that gives less mechanical stress on cells.

Few additional observations need to be remarked in order to make the model even closer to the common knowledge of the biology of vein graft:

- Macrophages in the wall typically getting access to the system from the lumen or through the vasa vasorum can be treated with the same particle mathematical framework, but with different parameters describing size, motility, et cetera.
- According to Peskin’s method (Peskin 2002), the IEL has a certain porosity that allows SMC to pass through according to the local mechanical stress of the membrane. This would be convenient since SMC can migrate from the media to the intima (Yuan et al. 2017; Hao et al. 2003).
- The volume of a “daughter” cell may increase in time to an average value after mitosis. In our implementation, a two-parameter description of the cell–cell interaction has been used, specifically one parameter for the cell side and one for the level of interaction, but in a more realistic representation the size of the cell should be time dependent.

Among all the potential improvements of the model, it is important to highlight how the level of detail is necessarily driven by how feasible is to retrieve specific biological measurements, needed to set up the model, from histological data. As an example, one should ask herself how challenging tracking macrophages or measuring cells dimensions at experimental level is. This should drive the always actual seek of a necessary compromise between accuracy and feasibility of the model.

Finally, most of the issues related to numerical stability and mass conservation of the IBM were not encountered because the flow is highly viscous with a low Reynolds number, and the membrane has relatively low tension in the immersed boundary calculation. Accordingly, there was no need to correct the scheme to ensure mass preservation as instead done in Pacull and Garbey (2009).

### Plan of simulations

After having described the framework of the model, a plan of the simulations is provided for the convenience of the reader, along with the details on their setup:

1. A **basic solution** needs to be retrieved in order to serve as baseline point for the vascular adaptation. The setup in order to reproduce a healthy vein at the time of implantation is the same already used in Garbey et al. (2017). It is important to remark how, due to the stochastic nature of the TP implementation, a result of the model can be considered as robust only if run a number of times sufficient to keep the standard deviation low, an aspect largely addressed in Garbey et al. (2017).
  As general rule within the presentation of the results, a graft’s cross section is chosen among *N* independent simulation as representative, while for the representation of the temporal dynamic of specific cellular events, the average trend is always chosen as the significant one.
2. **Intimal Hyperplasia** has been largely studied both by simulating its early phase, i.e. 1 day of adaptation, and by studying a longer follow-up like 1 month.
  The choice of running the model for different follow-up times is dictated by the need to distinguish the different cellular motility impacts during the early phase rather than during the late phase. Again the coefficients used to properly tune the probability laws described in Table 2 are the same already used to retrieve intimal hyperplasia in Garbey et al. (2017). A comparison between histology obtained from experimental data and the output of our model is also offered in order to appreciate the closeness to the experimental reality, especially in terms of variety of lumen encroachment.
3. A **cross-validation** between the new model and a zero-dimensional dynamical system developed by our group (Garbey and Berceli 2013) has been finally performed on a 4-month follow-up base as it has been also done for the ABM developed in Garbey et al. (2017). The motivation and the rationale are the same, and the DS has been run for cross-validation purposes with the following setup:
  A 50% decrease in shear stress from its baseline value to stimulate SMC migration and subsequent proliferation in intima.
  Initial graft (*R*)/lumen(*r*)/IEL (re) radii are set to be equal to the radii’s value recorded at the end of the basic solution generation for the PDE model, and they are $R = 0.3$; $\hbox {re} = 0.28$; $r = 0.24$ where all the measures are expressed in mm.
  The constant coefficient $\gamma$, which corresponds to $\alpha _5$, see Table 2 for reference, that leads SMC division within tunica intima represents the unknown coefficient that needs to be retrieved in order to cross-validate the two models.
  It is finally important to recall how, in order to calibrate the DS on the PDE model, the distance between the same output of the two models, that in this case is the temporal dynamic of intimal area on a 4-month follow-up, has been minimized by using a genetic algorithm (GA) and the optimum value of $\gamma$ retrieved accordingly.

## Results

### Basic solution

As anticipated in Methods section, the model was run in a steady-state condition in order to generate the basic solution representing the healthy vein at the time of implantation, here reported in Fig. 4a, where each red dot represents a SMC, while the green circle individuates the IEL. As it can be seen, the starting hypothesis was to occupy the wall with a 25% of SMCs while the remaining 75% is considered to be uniform ECM. Already the replication of the initial condition represents a good approximation of the histology of a vein graft, a detailed description of which is reported in Berceli et al. (2009).

**Fig. 5:**
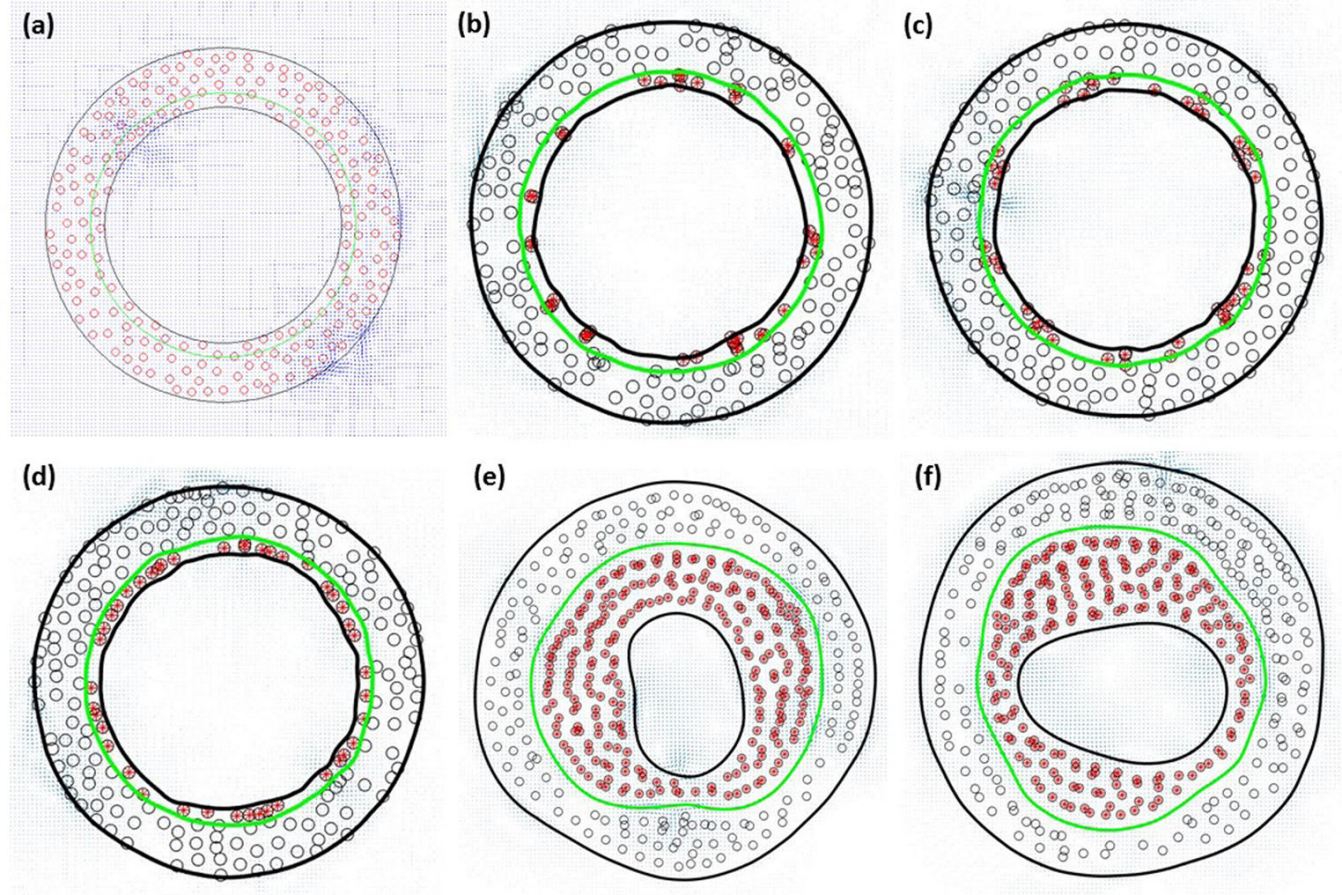
Simulations results Cross section of the vein graft is reported in **a** basic solution condition, i.e., healthy vein at the time of implantation, at early stage of hyperplasia progressively adding up, **b** random motion, **c** cell–cell repulsion, and **d** matrix invasion forces as cellular motility drivers. Finally, the late phase of hyperplasia is represented with an encroaching of the lumen affected by **e** vertical and **f** horizontal stretching of the lumen itself

### Intimal hyperplasia

The study of intimal hyperplasia on two different follow-up times is here presented in two separate subsections. As anticipated in Introduction, we systematically compared the cellular distribution obtained with our simulations with images from histology obtained from our experimental model of rabbit, a sample of which is shown in Fig. 5. It is useful to remark how the goal is here to be able to reach a relatively uniform distribution of cells within the intima and the media in order to be as close as possible to the character of distribution appreciable from Fig. 5.

**Fig. 6:**
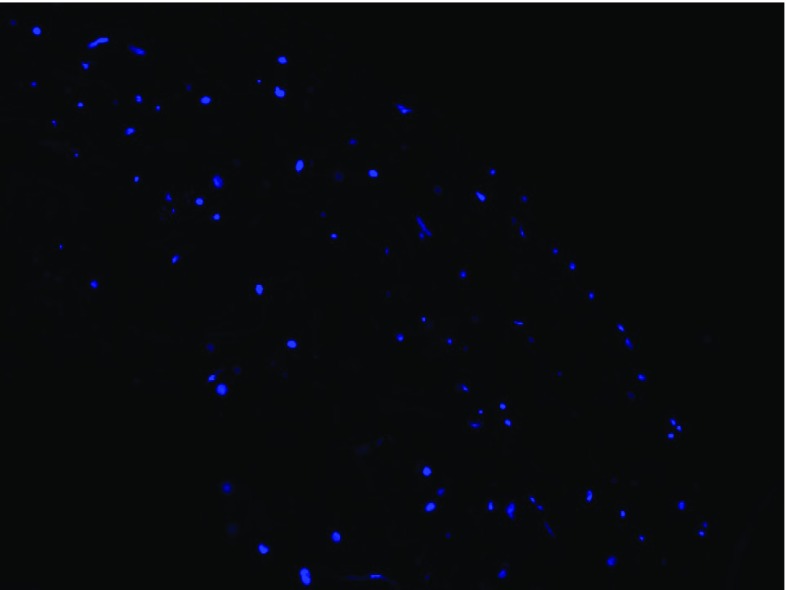
Histology of a cross section of vein graft staining image of a portion of graft’s wall where the blued dots identify the cells’ nuclei. The stack of images were obtained via confocal microscopy and post-processed in order to correct the artifacts due to the different depths of cells with respect to the plan of visualization

Having set this as standard to be reached, and being our hypothesis that this is feasible thanks to a detailed description of the forces driving the cellular motility, the analysis of the early stage of hyperplasia is here focused on verifying such hypothesis.

#### Early phase of hyperplasia

Figure 4b reports the results for the early phase of intimal hyperplasia showing how a random particles motion does not contribute to distribute the SMCs uniformly in the intima as instead retrievable from histology, and this is caused by the motion restriction that affects SMCs because of the initial thickness of the intima, which is about the dimension of a cell diameter.

Indeed, because the intima is so thin, if a cell undergoes apoptosis, it cannot be replaced by another one and the empty spot remains vacant. However, it is also true that by releasing this constraint on random SMC motion that exhibits friction to the wall, for example by using a very low stiffness for lumen wall or IEL, one allows a too large variation of the intima that collapses unrealistically in some points, while, also unrealistically, enlarges somewhere else.

By adding the repulsive cell–cell interaction, ranging up to two cells diameter, the distribution of SMCs within the intima gets more uniform as shown in Fig. 4c, even though one can still observe the formation of clusters of SMCs that will eventually be trapped in pockets of the lumen wall and there confined by the tension of the membrane itself.

In addition, from Fig. 4c, it is clear how some large areas of ECM with no SMCs have the tendency to form and it is evident how this deviates from the reality observed at histological data level, where SMCs distribution is more uniform in radial direction.

A more uniform distribution is reached by adding the matrix invasion term that is set up with an interaction coefficient $n_e=3$ as shown in Fig. 4d. The cross-analysis of Fig. 4b–d seems to confirm our hypothesis about the need of an accurate and detailed description of the potentials driving cellular motility, and this is not surprising seen that the reorganization of the wall plays in general a key role during the adaptation of the graft itself.

Finally, according to the purpose of the presented work, SMC proliferation was not activated in the tunica media and consequently a relatively stable and uniform distribution of SMCs within the media can be observed. More precisely, from the results presented in Fig. 4b–d, SMCs distribution in the media is not affected by the type of algorithm chosen to compute the cellular motility.

#### Late phase of hyperplasia

Figure 4e, f reports the result of two independent simulations run with a follow-up time of a month. It is interesting to observe how, on a longer follow-up, the SMC distribution still retains its asymmetric character, which is not clear whether it is justified at histological level or not.

Again, and not surprisingly, the distribution of the SMCs in the media remains relatively uniform and unvaried, which represents a solid milestone also considering that the new model is inherently a smaller ABM than the one presented in Garbey et al. (2017) but with a larger level of noise.

If needed, in order to promote radial symmetry, a potential solution will be to suppose that SMCs motility has a preferred motion in the direction orthogonal to the radius in order to align the cell arrangement with the dominant radial strain energy, or alternatively that cell proliferation is higher in areas characterized by a low SMCs density. Coupled to it, an increase in the relaxation time $\delta t$ might be another way to further foster the SMCs distribution toward the radial direction.

Figure 6 shows a nice comparison between histology data and our model. Data reported in the two left panels refer to rabbit experimental model where intimal hyperplasia was induced by graft ligation (Conte et al. 2006). It is nice to appreciate how our model can catch the different patterns of lumen encroachment, in this very case both horizontal and vertical invasion. This result is remarkable because it proves how our computational model shares the same uncertainty of a true experimental setup, making it suitable not only to test clinical hypotheses, but also to be used to design in advance an effective experiments to wisely choose the values of the driving coefficients of the model. An example is given in Casarin et al. (2018), where a computational model of graft adaptation is used in a twofold way: on the one end as virtual dataset generator and on the other end as a true computational model.

**Fig. 7:**
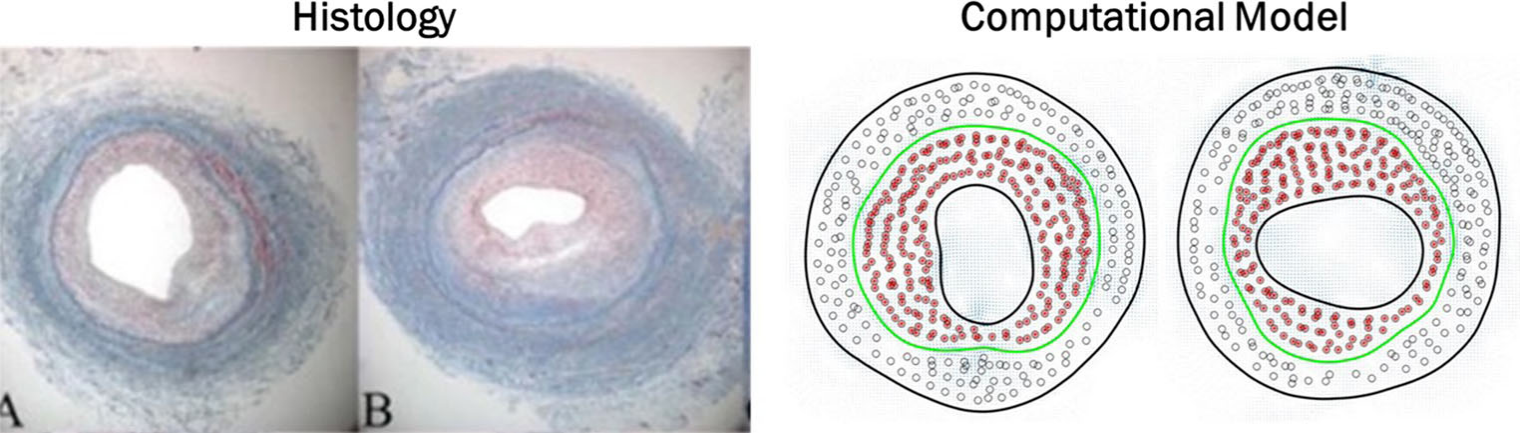
Histological comparison histology data of rabbit model setup to study intimal hyperplasia (left two panels) (Conte et al. 2006) are compared with the variety of graft’s cross sections obtained as output of our model (right two panels)

### Cross-validation

In order to cross-validate the DS and the current model, the very first step was to use the new PDE model to reproduce the qualitative patterns studied for intimal hyperplasia with our previous ABM (Garbey et al. 2017). The results of this step can be appreciated in Fig. 7, where the temporal dynamic of lumen area (a), intimal area (b), and medial area (c) are represented. In every panel, each independent simulation is marked with a different color and the average trend, which serves as the representative one, is marked with a bold black line.

**Fig. 8:**
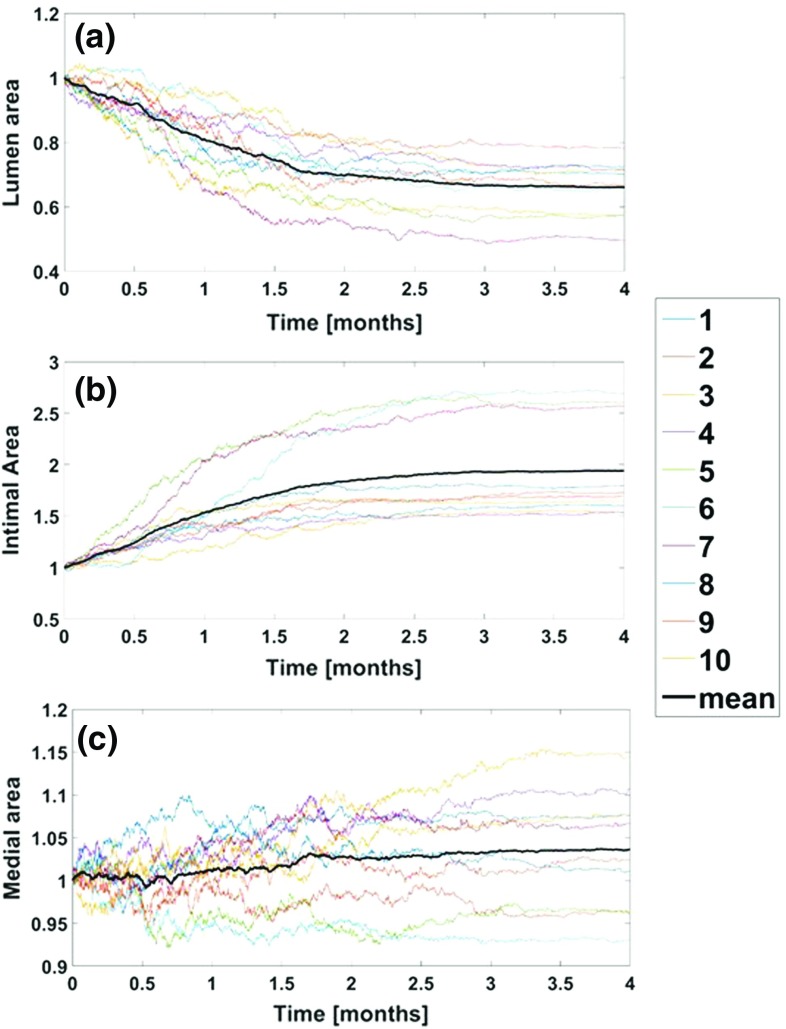
Intimal hyperplasia-long term follow up The temporal dynamics of **a** lumen area, **b** intimal area, and **c** medial area are represented on a postsurgical follow-up of 4 months. Each plot is normalized on the value recorded at the beginning of the follow-up. The colored lines correspond the output of a single simulation, while the black line corresponds to the mean trend of 10 different and independent runs

Finally, the result of the calibration, taking as output the temporal dynamic of lumen area, is reported in Fig. 8, showing a high level of accuracy with a percentile relative error lower than 2%, and requiring $\gamma$ to be set up equal to 4.6 in order to match with the DS the rate of intimal hyperplasia obtained with the PDE model.

**Fig. 9:**
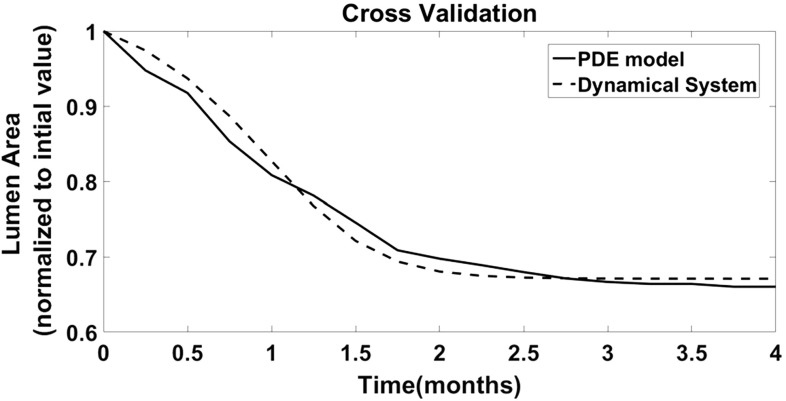
Cross-validation The Dynamical System is calibrated on the mean output of the PDE model. Figure shows the temporal dynamic of the lumen area as output of the PDE (solid line), and as output of the Dynamical System (dashed line) on a 4 months postsurgical follow-up

## Discussion and conclusion

A large stream of works on multiscale modeling of vascular adaptation, especially after stenting in arteries, has been so far developed, and it is available from the literature (Nolan and Lally 2018; Boyle et al. 2010; Zahedmanesh and Lally 2011; Zun et al. 2017; Amatruda et al. 2014; Tahir et al. 2013, 2014, 2015). Even though they do not take into account the detailed structure of the wall, they will serve as an excellent base to test the new generation of vascular treatments, such as covered stents or angioplasty using drug-eluted balloon. Taking inspiration from them, our model shares some of the main concepts and limitations of these multiscale models, even though our focus on vein graft adaptation, rather than arterial response to treatments, makes the requirements in terms of modeling very different. Arterial wall has high anisotropic properties as opposed to vein grafts because of the key role of ECM fibers in the media. During the intervention, the vein graft loses its adventitia, which is not the case for arteries, and during the early stage, the intimal layer is extremely thin and it undergoes through a dramatic adaptation. As discussed in Garbey and Berceli (2013), the interplay between the various components of the system, i.e., SMC, ECM, leads to a high complexity of feedback mechanisms that are usually well captured by the cited dynamical system.

It has been reported that the percentage of the volume of SMC in vein graft intima ranges from about 20–40% regardless of intimal thickness (Kohler et al. 1991; Kraiss et al. 1991; Zwolak et al. 1987). It is for this reason that a description where SMC get packed (Melnikova et al. 2017) may not be best in that situation.

It also seems essential to provide a computational model that can test various hypotheses on the role of the matrix reorganization and cell motility in vascular adaptation. Vein grafts are probably a good way to start because the detailed structure of the wall has less complexity than a main artery. However, the role of the matrix might have been underestimated (Evans et al. 2008) in most current computational models to some extent.

In the current work, a model of vascular adaptation has been implemented representing a generalization of a previous ABM developed by our group and laying on a hexagonal fixed grid structure.

Within this latter, the use of a fixed grid represented a clear limitation toward a model close enough to the physiological reality and it is for this reason that different techniques have been studied in order to bypass this kind of limitation (Hwang et al. 2009; Johnston et al. 2013; Browning et al. 2018).

In the new approach presented, we used a technique that relies almost entirely on PDEs and differential equations to compute the plasticity of the wall and the motility of the cells without being limited by a fixed grid. Delegating the goodness of the model almost entirely to the accuracy of the SMCs motion description made the thinking behind the architecture of the motion algorithm the real challenge of our approach.

As it has been appreciated in Results section, the key point was to understand which forces play a key role in providing a uniform cellular distribution across the wall. The power of the model is indeed its capability of testing different hypotheses at computational level in a short time and in an effective way.

One clear evidence retrieved by studying the output is that the matrix invasion by SMCs is pivotal in order to maintain mechanical homeostasis and consequently to reproduce experimental data accurately.

Another evidence is about the distance between cells; indeed, it has been proved both how a short distance interaction in the close proximity of a cell may not be sufficient to explain the evidences observed at histological level, but also how a distance of the order of few cells is enough to match the experimental reality, a statement supported by the concept of interstitial cellular flow of small molecules.

Future developments of the current model will certainly see the study of other cellular events, for example medial mitosis, ECM synthesis, and degradation both in intima and in media, cellular motility following inflammation during the very early postsurgical follow-up. These are only few of the many to be implemented in order to bring the model closer to the physiological reality. Also, a 3D extension will be implemented, which will set aside the uncertainties due to the low number of SMCs involved in each simulation. Another necessary future step will be represented by an extensive analysis of data from histology, in order to better reconstruct the initial structure of the vein, which will be done by following the experimental scheme proposed in Klein et al. (2017).

Finally, the work recently published by Browning et al. (2018), based on prostate cancer cell lines, gives an excellent example of what should come next in this vascular adaptation study.

### Electronic supplementary material

Below is the link to the electronic supplementary material.

Supplementary material 1 (pdf 209 KB)

## Appendix

### A1 Mechanical model

#### A1.1 Approximation of flow velocity and shear stress

In order to compute the steady potential flow inside the lumen, we used a zero-velocity boundary condition at the wall $\partial \varOmega _{\mathrm{lumen}},$ and the conservation of flux at the timescale of tissue plasticity, which is written as follows:

$$\begin{aligned} \int _{\varOmega _{\mathrm{lumen}}} u \; \hbox {d}y \; \hbox {d}z = \mathrm{Flux}, \end{aligned}$$

Further, the flow inside the lumen is driven by a constant pressure gradient $\frac{\hbox {d}P}{\hbox {d}x},$ as shown in Figure S1. From this potential flow solution, the equations for the fully developed duct flow are written as

$$\begin{aligned} \frac{d^2 u}{\hbox {d} y^2} + \frac{d^2 u}{\hbox {d} z^2} = \frac{1}{\mu } \; = \frac{\hbox {d}P}{\hbox {d}x} = C^t, u_{| \partial \varOmega _{\mathrm{lumen}}} =0, \end{aligned}$$

and they are used to compute the normal shear stress component at the wall ($\tau _{\mathrm{wall}}$), where $\mu$ is viscosity. A finite volume scheme and an immersed boundary implementation of the nonslip condition at the wall are then used to compute the fluid velocity on a regular spatial grid. While fluid shear stress is applied only on the luminal surface $\partial \varOmega _{\mathrm{lumen}}$, the biological effect of this shear stress [e.g. growth factor (*G*) and cytokine production] is transferred to the most superficial portions of the wall solver.

#### A1.2 Analytical model of thick wall tissue deformation

This model can be used at the early stage of the vein graft adaptation when the graft is close to radial symmetry (Zhao et al. 2003):

The wall deformation depends on the difference between pressure at lumen wall and the pressure at the EEL wall, defined as

$$\begin{aligned} \Delta P = P_{\mathrm{EEL}}-P_{\mathrm{lumen}}. \end{aligned}$$

The transmural pressure $\Delta P$ generates the load. Let us assume first that the vein graft has a thick wall $(R_{\mathrm{lumen}}, R_{\mathrm{EEL}})$ with uniform linear elastic mechanical properties. For simplicity, we make the hypothesis of an open-ended tube, i.e., $\sigma _z=0.$ The radial stress $\sigma _r$ and circumferential stress $\sigma _{\theta }$ are then given by Kleinstreuer (2006),

$$\begin{aligned} \sigma _r(r)=\frac{p_1 r_1^2}{r_2^2-r_1^2} \; \left( 1-\frac{r_2^2}{r^2}\right) \; - \; \frac{p_2 r_2^2}{r_2^2-r_1^2} \; \left( 1-\frac{r_1^2}{r^2}\right) , \end{aligned}$$

and

$$\begin{aligned} \sigma _{\theta }(r)=\frac{p_1 r_1^2}{r_2^2+r_1^2} \; \left( 1-\frac{r_2^2}{r^2}\right) \; - \; \frac{p_2 r_2^2}{r_2^2+r_1^2} \; \left( 1-\frac{r_1^2}{r^2}\right) , \end{aligned}$$

where $$r_1=R_{\mathrm{lumen}}, \; r_2=R_{\mathrm{EEL}}, \; p_1=P_{\mathrm{lumen}}, \; p_2=P_{\mathrm{EEL}}.$$

The stress distribution in the radial symmetric thick cylinder case is independent on the stiffness or compression ratio of tissue, i.e., no dependence on Young’s modulus *E* neither Poisson ratio $\nu .$ is recorded. This is, however, not true for the displacement which is so defined:

$$\begin{aligned} u(r)=\frac{C_1}{2} \; r + \frac{C_2}{r}, \end{aligned}$$

where

$$\begin{aligned} C_1 \; (\lambda + \mu )= & {} \frac{p_1 r_1^2 - p_2 r_2^2 }{r_2^2-r_1^2},\\ 2 \; \; \mu C_2= & {} \frac{p_1 - p_2 }{r_2^2-r_1^2} \; r_1^2 \; r_2^2, \end{aligned}$$

and $\lambda ,\mu$ are the Lamé coefficients:

$$\begin{aligned} \lambda = \frac{E \nu }{(1+\nu )(1-2\nu )}, \quad \mu =\frac{E}{2(1+\nu )}. \end{aligned}$$

#### A1.3 FE model of thick wall tissue deformation

If the wall looses radial symmetry, one must use a numerical algorithm to retrieve the wall tension at specific locations inside the wall. It is convenient then to use a FE code based on a more elaborate model of tissue deformation than just a simple linear elasticity model.

We used a neo-Hookean model to find the deformation of tissue of the vein:

$$\begin{aligned} W = \mu /2 \; \left( J^{-\frac{2}{3}} \; I_1 -3\right) + \; \frac{K}{2} \; (J-1)^2, \end{aligned}$$

where *W* is the strain energy per unit of volume, $I_1$ is the first invariant of the left Cauchy–Green deformation tensor, $\mu$ is the initial shear modulus of the material, *K* is the bulk modulus, and *J* is the determinant of the elastic deformation gradient.

We use the software Gmsh to generate the finite element mesh of the wall and Febio software (Maas et al. 2012) to calculate the tension at each node of the mesh after applying neo-Hookean model:

$$\begin{aligned} \sigma = \sqrt{[(\lambda _1-\lambda _2)^2+(\lambda _2-\lambda _3)^2+(\lambda _3-\lambda _1)^2]/2}. \end{aligned}$$

where $\lambda _i$ are the eigenvalues of the stress or strain tensor of each point of the node. We refer to Garbey et al. (2015) for a detail description of the implementation.

### A2 Agent-based model

The agent-based model that describes the SMC mitosis and apoptosis as well as ECM deposition/degeneration follows the algorithm given in Figure S2, while Table 2 provides the probability laws for each cellular event.

### A3 IBM algorithm

The primitive variables are *V* and *P*, respectively, the velocity and pressure of the fluid, which physical parameters correspond to the uniform viscosity $\mu$ and the uniform density $\rho$. The fluid domain $\varOmega =(0,1)^2 \subset \mathbb {R}^2$ is a square described by the Cartesian coordinate vector *x*. $\varGamma \subset \varOmega$ is denoted as a generic immersed elastic boundary, which curvilinear dimension is *m* ($m \le d$). *X* is the Lagrangian position vector of $\varGamma$, expressed in the *d*-dimensional Cartesian referential. The Lagrangian vector *f* is the local elastic force density along $\varGamma$, also expressed in the Cartesian referential. *f* is projected onto $\varOmega$ to get the Eulerian vector field *F* that corresponds to the fluid force applied by the immersed elastic boundaries.

For simplicity, the method is here restricted to a single immersed elastic boundary, even though in order to fit the anatomy of the vein graft, see Fig. 4, three separate immersed boundaries have been used: (i) the lumen wall $\varGamma _{\mathrm{lumen}}$, (ii) the IEL $\varGamma _{\mathrm{IEL}}$, and the external elastic lamina (EEL) $\varGamma _{\mathrm{EEL}}.$

If $s\in (0,1)^m$ is the curvilinear coordinate of any points along $\varGamma$, and $t\in [0,t_{\mathrm{max}}]$ is the time variable, the different mappings can be summarized as follows:

$$\begin{aligned} V:(x,t) \in \varOmega \times [0,t_{\mathrm{max}}]\longrightarrow & {} \mathbb {R}^2\\ P:(x,t) \in \varOmega \times [0,t_{\mathrm{max}}]\longrightarrow & {} \mathbb {R}\\ X:(s,t) \in (0,1)^m \times [0,t_{\mathrm{max}}]\longrightarrow & {} \varOmega \\ f:(s,t) \in (0,1)^m \times [0,t_{\mathrm{max}}]\longrightarrow & {} \mathbb {R}^2\\ F(x,t) \in \varOmega \times [0,t_{\mathrm{max}}]\longrightarrow & {} \mathbb {R}^2 \end{aligned}$$

One of the cornerstones of this method is the explicitation of the fluid–elastic interface interaction, which model is unified into a set of coupled partial differential equations (PDEs). To build that, the incompressible Navier–Stokes system is written as:

7 $$\begin{aligned} \rho \left[ \frac{\partial V}{\partial t} + (V.\nabla )V \right]= & {} -\nabla P + \mu \Delta V +F \end{aligned}$$

8 $$\begin{aligned} \nabla \cdot V= & {} 0 \end{aligned}$$

The IBM algorithm requires the extrapolation of the Lagrangian vector *f* into the Eulerian vector field *F* from the right end side of (7). For this purpose, a distribution of Dirac delta functions $\delta$ is used, such as:

9 $$\begin{aligned} F(x,t)= & {} \int _{\varGamma } f(s,t) \delta \left( x-X(s,t) \right) \nonumber \\ \hbox {d}s= & {} {\left\{ \begin{array}{ll} f(s,t), &{} \quad \text {if} \; \; x=X(s,t)\\ 0, &{} \quad \text {otherwise} \end{array}\right. } \end{aligned}$$

The motion of the immersed boundary finely mimics the motion of the particles suspended in a fluid thanks to the nonslip boundary condition, approximated with (10) by using the Dirac delta function as an interpolating tool for *V*, between $\varOmega$ and $\varGamma$:

10 $$\begin{aligned} \frac{\partial X(s,t)}{\partial t}= & {} \int _{\varOmega } V(x,t) \delta (x-X(s,t)) \nonumber \\ \hbox {d}x= & {} {\left\{ \begin{array}{ll} V(X(s,t),t), &{} \quad \text {if} \; \; x=X(s,t)\\ 0, &{} \quad \text {otherwise} \end{array}\right. } \end{aligned}$$

The immersed boundary dynamic is regulated with a linear elastic model that has been implemented by using the Hooke’s law of elasticity, for which the tension $\mathcal {T}$ of the immersed boundary is a linear function of the strain energy. For a one-dimensional boundary like the one being described, the tension is written as

11 $$\begin{aligned} \mathcal {T}(s,t)=\sigma \left| \frac{ \partial X(s,t) }{ \partial s } \right| , \end{aligned}$$

where $\sigma$ is the boundary elasticity coefficient, and consequently the local elastic force density *f* is written as

12 $$\begin{aligned} f(s,t) = \frac{\partial \left( \mathcal {T}(s,t) \tau (s,t) \right) }{\partial s}, \quad \tau (s,t)= \frac{\partial X(s,t)/ \partial s}{\left| \partial X(s,t)/ \partial s \right| }, \end{aligned}$$

with $\tau$ being the vector tangent to $\varGamma$. Finally, by replacing (11) into (12), the local elastic force density assumes its final form that is written as

13 $$\begin{aligned} f(s,t)=\sigma \frac{\partial ^2 X(s,t)}{\partial s^2}. \end{aligned}$$
