# Supplementary material for: A versatile hybrid agent-based, particle and partial differential equations method to analyze vascular adaptation
Source: Biomech Model Mechanobiol. 2018 Aug 9;18(1):29–44. doi: 10.1007/s10237-018-1065-0 (PMC6373284; doi:10.1007/s10237-018-1065-0)
Supplement: Supplementary file 1 — Supplementary material 1 (pdf 209 KB) [file 10237_2018_1065_MOESM1_ESM.pdf]

Supplemental Figures

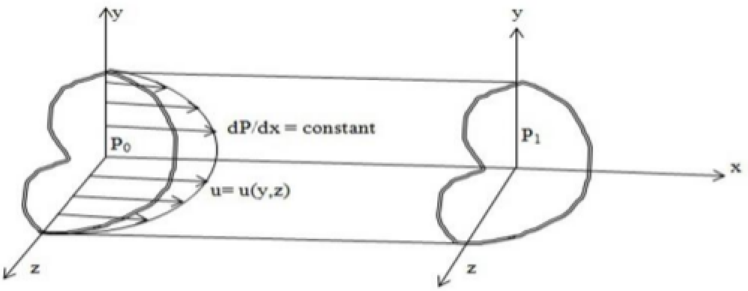

Figure S1. Fully developed flow through the lumen.

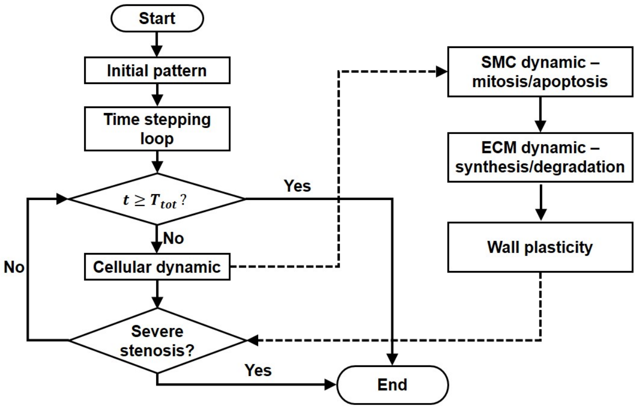

Figure S2. ABM Algorithm: an intial pattern is created by assigning to each site of the ABM either an SMC or an ECM, also combined with its corresponding internal clock time. Next, each site undergoes either mitosis or apoptosis according to the axiomaic rules described in Table 2 to then get re-distributed via tissue remodeling algorithm (Wall Plasticity) described in this paper in Section 2.3 [21].
